# Subacute thyroiditis during early pregnancy: a case report and literature review

**DOI:** 10.1186/s12884-021-04368-2

**Published:** 2022-01-07

**Authors:** Chao-Fang Bai, Guang-Hui Shen, Ying Yang, Ke Yang, Melvin R Hayden, Yuan-Yuan Zhou, Xing-Qian Geng

**Affiliations:** 1 Department of Endocrinology, Affiliated Hospital of Yunnan University, Second People’s Hospital of Yunnan Province, Kunming, 650000 Yunnan Province China; 2 Institute of Pediatrics of Children’s Hospital of Fudan University, Shanghai, 201102 China; 3 Department of Vascular and Cardiology, Ruijin Hospital, Shanghai Jiaotong University of Medicine, Shanghai, 201102 China; 4 University of Missouri School of Medicine, Departments of Internal Medicine, Endocrinology Diabetes and Metabolism, Diabetes and Cardiovascular Disease Center, Columbia, MO USA; 5 Department of Endocrinology, The Sixth Affiliated Hospital of Kunming Medical University, Yuxi, 650031 Yunnan Province China

**Keywords:** Subacute Thyroiditis, Pregnancy, Hormones, Hyperthyroidism, Case report

## Abstract

**Background:**

Subacute thyroiditis (SAT) is rarely diagnosed in pregnant women, and only 7 cases have been reported to date. Thyroid dysfunction, especially hyperthyroidism, during pregnancy has been associated with both maternal and neonatal complications. Thus, the early diagnosis and treatment of SAT during pregnancy may be beneficial. We present a case report and literature review to complement the diagnostic evaluation and management of SAT during pregnancy.

**Case presentation:**

A 27-year-old woman presented in gestational week 17 of her first pregnancy and had a negative prior medical history. She presented to the Endocrinology Department complaining of neck pain for one month that had intensified in the last five days. Physical examination revealed a diffusely enlarged thyroid gland that was firm and tender on palpation. The patient also had an elevated temperature and heart rate. The increasing and long-lasting pain coupled with a decreased level of thyroid-stimulating hormone indicated hyperthyroidism. Ultrasound findings were indicative of SAT. Importantly, the pain was so severe that 10 mg of oral prednisone per day was administered in gestational week 18, which was increased to 15 mg/d after 10 days that was discontinued in week 28. Levothyroxine was started in gestational week 24 and administered throughout the pregnancy. The patient responded well to the treatments, and her neck pain disappeared in gestational week 21. She gave birth to a healthy male in gestational week 41.

**Conclusion:**

SAT can be diagnosed and effectively managed during pregnancy, thus benefiting mothers and infants.

## Background

The incidence of maternal thyroid disorders remains high during pregnancy, with hypothyroidism affecting up to 2%—3% of all pregnancies and hyperthyroidism affecting 0.1%—0.4%. Subacute thyroiditis (SAT) accounts for 5% of patients with thyroid disease [1], Pregnant women with clinical and subclinical hypothyroidism increased the risk of preterm delivery by 4.4 times—3.0 times respectively. Clinical hyperthyroidism is also significantly associated with fetal distress [2]. SAT is also called granulomatous thyroiditis or giant cell thyroiditis, and is regarded as a self-limiting inflammatory disorder.

The majority of SAT patients are women between the ages of 30 and 50 years. SAT is secondary to virus infection and is frequently accompanied by neck pain, fever, fatigue, and myalgia, followed by diffuse enlargement and tenderness of the thyroid gland [3, 4]. Laboratory examination tends to show an increase in the level of erythrocyte sedimentation rate (ESR), white blood cell count, C-reactive protein (CRP), and other indicators of infection, coupled with the low echogenicity of nodules on ultrasound and decreased iodine absorption rate. SAT in pregnancy is extremely rare and is frequently misdiagnosed as hyperthyroidism. While 25% of patients have hyperthyroidism in pregnancy [5], hyperthyroidism caused by SAT during pregnancy is uncommon, with only 7 cases reported to date [5–10].

Herein, we present a case of SAT diagnosed in the first trimester of pregnancy in the Endocrinology Department of the Second People’s Hospital of Yunnan Province (Yunnan, China).

### Case presentation

A 27-year-old women in the 17^th^ week of gestation in her first pregnancy presented to the Endocrinology Department of our hospital complaining of neck pain for one month that had intensified in the last five days. The neck pain appeared at the 12^th^ week of gestation, which was on the left side without any obvious cause and radiated to the jaw. It was initially tolerable, and there were no other symptoms, such as chills, fever, palpitations, tremor, or sweating. The pain gradually shifted to the right side and the patient requested no medication for her discomfort. In the 17^th^ week of gestation, the patient suffered from severe neck pain, which seriously affected quality of life and sleep and she expressed a strong willingness to undergo treatment.

The patient had throat pain for 3 days before pregnancy, which improved without treatment and had no personal or family history of thyroid disease. Her menstrual cycle was regular before pregnancy. She had an axillary temperature of 37.4 °C, increased resting heart rate of 98 beats/min, and normal blood pressure of 110/70 mmHg in our hospital. Physical examination revealed bilateral enlargement of the thyroid gland that was firm and tender to palpation.

**Fig. 1:**
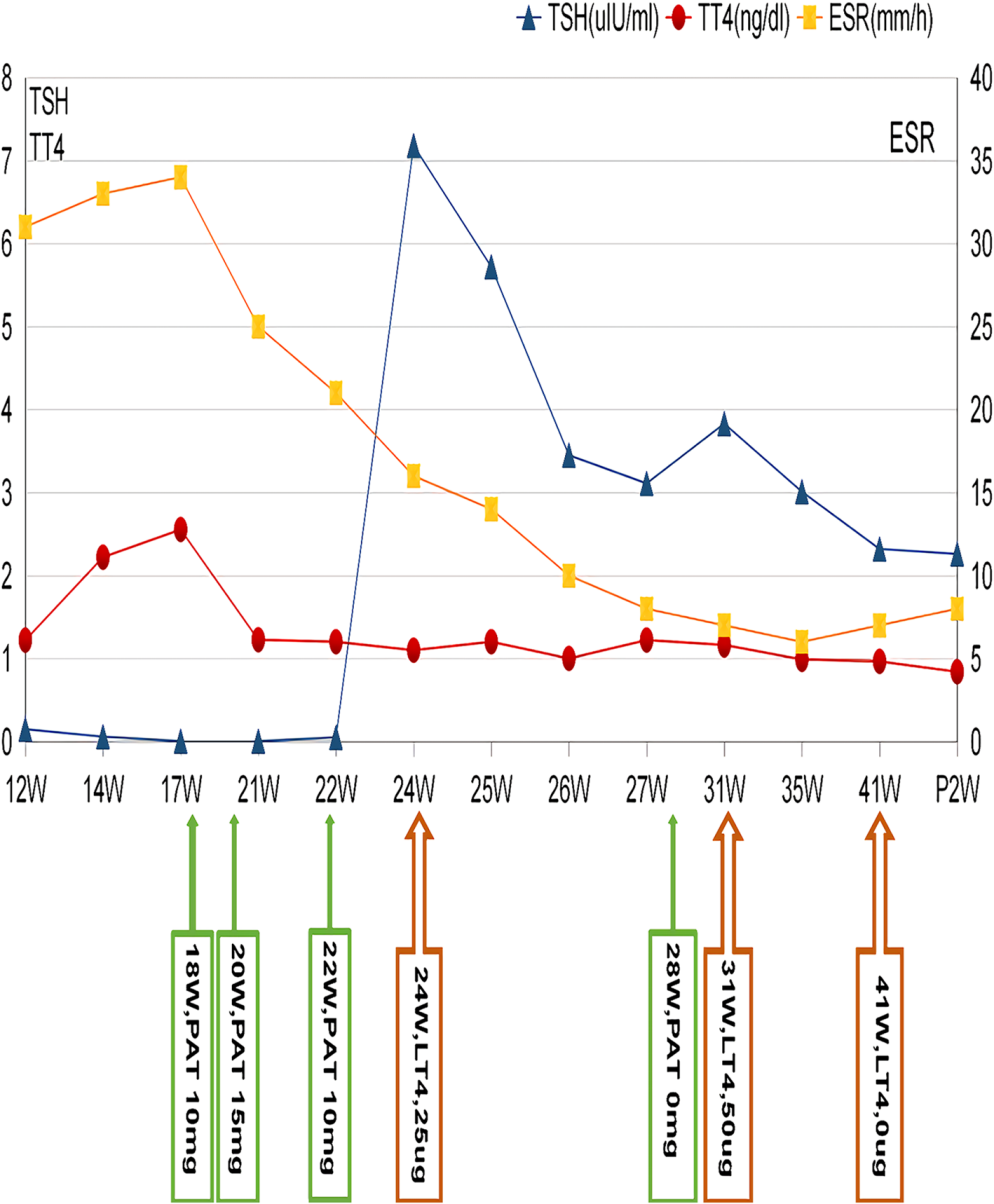
Changes in thyroid function and erythrocyte sedimentation rate. *ESR*: Erythrocyte sedimentation rate; *LT4*: Levothyroxine; *P*: Postpartum; *PAT*: Prednisone Acetate Tables; *TSH*: Thyroid-stimulating hormone; *TT4*: Thyroxine; *W*: Weeks of gestation

Thyroid function test results in the 12^th^ week of gestation showed the following: serum thyroxine (T4) 122.168 ng/mL (reference range: 50–130 ng/mL); serum triiodothyronine (T3) 1.481 ng/mL (reference range: 0.8–1.9 ng/mL); serum-free thyroxine (FT4) 9.444 pmol/L (reference range: 9.1–24.8 pmol/L); serum-free triiodothyronine (FT3) 4.321 pmol/L (reference range: 3.3–9.15 pmol/L); serum thyroid-stimulating hormone (TSH) 0.149 ulU/mL (reference range: 0.27–4.2 uIU/mL); anti-thyroglobulin antibody < 6% (reference range: < 30); thyroid peroxidase antibody < 5% (reference range: < 34); thyrotropin receptor antibody 4.3 U/L (reference range: < 12); and, ESR 31 mm/h (reference range: 0–20 mm/h) (Table 1, Fig. 1). Thyroid ultrasound showed that the solid area of the left thyroid lobe had low echogenicity, suggesting SAT. Thyroid function test results in the 14^th^ week of gestation were as follows: T4 222.1 ng/mL; T3 3.07 ng/mL; and, TSH 0.058 ulU/mL (Table 1, Fig. 1). Her thyroid function was checked again in the 17^th^ week and the results showed T4 255.4 ng/mL, T3 3.18 ng/mL, and TSH 0.005 ulU/mL( Table 1, Fig. 1).

**Table 1:** Laboratory data of thyroid function and erythrocyte sedimentation rate

Week of gestation	TSH (0.27–4.2 uIU/mL)	TT4 (50–130 ng/mL)	TT3 (0.8–1.9 ng/mL)	FT4 (9.1–24.8 pmol/L)	FT3 (3.3–9.15 pmol/L)	TPOAB (< 34 IU/ml)	TG (< 25 ng/ml)	ESR (0-20 mm/h)
12 W	0.149	122.168	1.481	9.444	4.321	5	6.1	31
14 W	0.058	222.1	3.07	21.33	5.7	—	—	33
17 W	0.005	255.4	3.18	21.45	6.02	8.74	—	34
21 W	0.005	122.63	1.7	21.14	5.71	8.07	15.28	25
22 W	0.049	120.23	1.76	22.04	3.9	7.54	—	21
24 W	7.18	110.02	1.81	14.46	4.59	14.01	15.72	16
25 W	5.72	120.23	1.86	14.27	5.27	13.23	19.72	14
26 W	3.45	100.15	1.67	13.97	5.46	20.74	—	10
27 W	3.11	122.25	1.83	12.76	5.27	13.92	—	8
31 W	3.83	116.43	1.8	16.44	7.08	8.44	14.89	7
35 W	3.01	98.92	1.73	13.92	5.08	10.25	9.48	6
41 W	2.32	96.54	1.86	12.64	4.8	8.88	—	7
P2W	2.26	84.04	1.38	11.32	4.95	19.83	14.52	8

*W* Weeks of gestation, *P* Postpartum, *TSH* Thyroid-stimulating hormone, *TT4* Thyroxine, *TT3*:Triiodothyronine, *FT4* Serum-free thyroxine, *FT3* Serum-free triiodothyronine, *TPOAB* Thyroid peroxidase antibody, *TG* Thyroglobulin, *ESR* Erythrocyte sedimentation rate

Based on patients symptoms, physical examination and laboratory findings she was diagnosed with SAT. Topical hydrocortisone ointment was given to alleviate the neck pain; however, she developed a fever after 2 days, which peaked at 37.8 ºC, and unbearable neck pain. The pain was so severe that 10 mg per day oral prednisone was administered, which was increased to 15 mg per day 10 days later for unalleviated pain. The patient responded well to the treatment and she was really grateful for her mitigation of her pain. The neck pain disappeared in the 21^th^ week of gestation. The thyroid function test results were as follows: T4 122.63 ng/mL; T3 1.7 ng/mL; and TSH 0.005 ulU/mL (Table 1, Fig. 1). In the 24^th^ week, the results were: T4 110.02 ng/mL; T3 1.81 ng/mL; and TSH 7.18 ulU/mL (Table 1, Fig. 1). Meanwhile, 25 ug levothyroxine was also administered, starting in the 24^th^ week of gestation and was continued throughout the pregnancy. The thyroid function and ESR were normal in the 27^th^ week of gestation (Table 1, Fig. 1) and therefore,prednisone treatment was stopped in the 28^th^ week but levothyroxine was continued. Thyroid function in gestational week 31 showed that TSH level was 3.83 ulU/mL (Table 1, Fig. 1). Therefore, the dose of levothyroxine was increased to 50 ug per day until delivery. Throughout this time the patient’s TSH level was maintained at 2.26–3.01 ulU/mL (Table 1, Fig. 1).

Glucocorticoid levels are high during pregnancy due to an increase in estrogen in the circulation, which promotes the generation of glucocorticoid-binding globulin and increases corticosteroid hormones in the plasma [11]. Importantly, extensive low echogenicity seen on ultrasound is also an essential and sufficient indicator for glucocorticoid use [5]. The patient in this study as well as three reported SAT cases during pregnancy were treated with glucocorticoids, which effectively alleviated the pain of patients [5, 7, 10].

While discontinuing to take her levothyroxine replacement at the 41^th^ week of gestation, the patient gave birth to a healthy baby boy via caesarean section, with a weight of 3600 g and an Apgar score of 9–10. Thyroid function test 2 weeks postpartum were as follows: TSH 2.26 ulU/mL; T4 84.04 ng/mL; and, T3 1.38 ng/mL (Table 1, Fig. 1).

## Discussion and conclusion

In general,one or two women suffer from thyrotoxicosis per 1000 pregnancies. Hiraiwa assumed that SAT accounts for 1% of thyrotoxicosis, 10–20 out of one million pregnant women will develop SAT [5]. In this study, we report a case of a pregnant woman with SAT, which is very uncommon,To date, only seven cases have been reported [5–10] (Table 2). Therefore, there is a need to carefully manage such cases.

**Table 2:** Summary and review of case reports in pregnant women with subacute thyroiditis

Case report time	Age	Time of SAT	Symptoms	Physical examination of thyroid gland	Thyroid ultrasound	Treatment	Delivery time	Newborn weight(g)	Apgar score	Postpartum thyroid function test results
Case 1 (year 2006)	35Y	11 W	fever, tired, Neck pain	enlarged thyroid gland and tender on plapation	Irregular hypoechoic zone was find in thyroid gland with irregular edges	Prednisone 20 mg per day, halved after 2 weeks; Liothyronine 10ug per day at 27 weeks of pregnancy, L-T4 50ug per day at 29 weeks until delivery	41 W	3968	10,10	Not provided
Case 2 (year 2006)	31 Y	6 W	Neck pain	enlarged right thyroid gland, firm and tender on plapation	Irregular hypoechoic thyroid gland on the right, 41 days later, the ultrasound findings on the left are as same as those on the right	Untreated	42 W	3265	9,10	Not provided
Case 3 (year 2010)	30 Y	6 W	Neck pain, tired	enlarged thyroid gland and tender on plapation	Irregular hypoechoic zone was find in thyroid gland with irregular edges	L-T4 50-75ug per day at 14 weeks of pregnancy	39 W	2720	9,10	Normal range
Case 4 (year 2012)	28 Y	9 W	Neck pain, tired	enlarged thyroid gland, firm and tender on plapation	The border of the right thyroid gland is not clear and the texture is uneven	L-T4 50ug per day at 20 weeks of gestation, 62.3ug per day after 3 weeks, and gradually reduce the dose to stop taking the drug after delivery	40 W	3400	10,10	Normal range
Case 5 (year 2012)	29 Y	5 W	unbearable to heat, palpitations, Neck pain	The right thyroid gland is enlarged slightly	Decreased in blood flow	Prednisone 1 mg/kg per day, stop taking prednisone after 2 months and oral L-T4 in dose of 2 µg/kg per day, increase to 2.4 µg/kg per day after 6 weeks, stop in 1 month postpartum	Not provided	Not provided	Not provided	Not provided
Case 6 (year 2015)	33 Y	13 W	Neck pain and tumefaction, tired	enlarged thyroid gland and tender on plapation	Not provided	Acetaminophen 3 × 500 mg per day, stop after 10 days and supplement L-T4 50ug per day, increase to 75ug per day after 3 weeks until delivery	38 W	3740	Not provided	Normal range
Case 7 (year 2015)	29 Y	5 W	Neck pain, tired, severe nausea and vomiting	enlarged thyroid gland	Abnormal thyroid texture	Prednisone 20 mg per day, pregnancy was terminated due to severe nausea and vomiting at 11 weeks	_	_	_	_
The case (year 2020)	27 Y	12 W	Unbearable neck pain	enlarged thyroid gland, firm and tender on plapation	Reduced in thyroid echo and texture was uneven	Topical hydrocortisone cream for neck, oral prednisone 10 mg per day after 2 days, increase to 15 mg per day after 10 days, stop at 27 weeks of gestation; L-T4 25ug per day at 25 weeks of gestation, increase to 50ug per day at 31 weeks of gestation to delivery	41 W	3600	9, 10	Normal range

*Y* years old, *W* Weeks of gestation

While the pathogenesis of SAT remains unknown.However, it has been postulated that viral infection leads to the production of an antigen that binds tightly to the human leukocyte antigen-B35 molecule on macrophages, activating cytotoxic T lymphocytes through helper T1 cells (Th1 cells). With the destruction of thyroid follicular cells, thyroid hormone is released into the blood, which results in symptoms of thyrotoxicosis [12]. Further, the hypofunction of iodine upake appears as thyroid follicular cells are destroyed, and the depletion of thyroid hormone is accompanied by hypothyroidism. Most thyroid functions return to normal as patients recover from SAT [9]. During pregnancy, B lymphocytes are inhibited while Th2 cells are activated, which dampen the function of Th1 cells. This may partially explain the low incidence and insignificant clinical symptoms of SAT during pregnancy [13].

SAT diagnosis is based on the patient’s medical history, symptoms, physical and laboratory fingings along with exclusion of other reasons for hyperthyroidism. Our patient presented with SAT symptoms in the 12^th^ week of gestation, in accordance with previously published case reports of SAT during early pregnancy (sixth to 13^th^ week) [5–10]. It is important to recognize thyroid dysfunction caused by human chorionic gonadotropin (HCG) in the first trimester. HCG levels increase significantly in early pregnancy and its structure is homologous to TSH, which leads to a transient rise in free thyroxin (FT4) and suppression of TSH; however, there is no neck pain during normal early pregnancy [13].

The physical findings of neck pain favors SAT in the differential diagnosis of SAT from other causes of hyperthyroidism or hypothyroidism during pregnancy. However, in some patients with Hashimoto’s thyroiditis [14], thyroid cancer or primary thyroid lymphoma, or even Graves’ disease may also have neck pain and tenderness of the thyroid gland in some cases. Importantly, all of the previously reported 7 patients with SAT during pregnancy had neck pain. Pain from SAT can be initially confined to or begin from the lateral gland, and then transferred to the contralateral gland [9]. In our case, the patient’s neck pain was found to be transferable and consistent with SAT. Importantly, additional clinical symptoms, such as fever and fatigue, were also observed.

Hyperthyroidism during SAT in pregnant women, manifested as low TSH and increased T3 and T4, needs to be differentiated from Graves’ disease. SAT patients also have a high ESR or CRP but poor radionuclide uptake in thyroid scintigraphy, although fine-needle aspiration biopsy may also be considered as a diagnostic method [13]. Howerver, pregnant women are not recommended to undergo thyroid scintigraphy and fine-needle aspiration biopsy has a higher inherent risk, which may contribute to the challenge of diagnosing pregnancy complicated with SAT..SAT is characterized by a low ratio of TT3 (ng/dL) to TT4 (µg/dL) < 20, FT3 to FT4 < 0.3 [15] and peripheral-blood eosinophil/monocyte (Eo/Mo) ratio < 0.2 [16]. These indicators are valuable for distinguishing SAT from Graves’ disease. In this case, TT3/TT4 = 12.12, FT3/FT4 = 0.46, and Eo/Mo = 0.076, consistent with the literature [15, 16], with the exception of FT3/FT4.

The diagnosis of SAT in pregnancy is based on the patient’s medical history, symptoms, physical and laboratory findings and exclusion of other reasons for thyroid dysfunction. Monitoring of the changes in thyroid hormone may also play an important role in the treatment of SAT.

## Data Availability

The datasets analyzed during the current study are not publicly available due to protection of the patient’s privacy but are available from the corresponding author on reasonable request (email: yangying2072@126.com).

## Abbreviations

SAT: Subacute thyroiditis
ESR: Erythrocyte sedimentation rate
CRP: C-reactive protein
T4: Thyroxine
T3: Triiodothyronine
FT4: Serum-free thyroxine
FT3: Serum-free triiodothyronine
TSH: Thyroid-stimulating hormone
HCG: Human chorionic gonadotropin
