# Correction to: Subacute thyroiditis during early pregnancy: a case report and literature review

**DOI:** 10.1186/s12884-022-04390-y

**Published:** 2022-01-31

**Authors:** Chao-Fang Bai, Guang-Hui Shen, Ying Yang, Ke Yang, Melvin R. Hayden, Yuan-Yuan Zhou, Xing-Qian Geng

**Affiliations:** 1 Department of Endocrinology, Affiliated Hospital of Yunnan University, Second People’s Hospital of Yunnan Province, Kunming, 650000 Yunnan Province China; 2 Institute of Pediatrics of Children’s Hospital of Fudan University, Shanghai, 201102 China; 3 Department of Vascular and Cardiology, Ruijin Hospital, Shanghai Jiaotong University of Medicine, Shanghai, 201102 China; 4 Departments of Internal Medicine, Endocrinology Diabetes and Metabolism, Diabetes and Cardiovascular Disease Center, University of Missouri School of Medicine, Columbia, MO USA; 5 Department of Endocrinology, The Sixth Affiliated Hospital of Kunming Medical University, Yuxi, 650031 Yunnan Province China

**Correction to: BMC Pregnancy Childbirth 22, 19 (2022)**

**https://doi.org/10.1186/s12884-021-04368-2**

Following publication of the original article [1], one of the authors was mistakenly deleted in the proof. The complete author names are listed below:

Chao-Fang Bai^1,†^, Guang-Hui Shen^2,†^, Ying Yang^1,*^, Ke Yang^3^, Melvin R. Hayden^4^, Yuan-Yuan Zhou^1,5^, and Xing-Qian Geng^1^

The original article has been corrected.
